# Latent class growth mixture modeling of HbA1C trajectories identifies individuals at high risk of developing complications of type 2 diabetes mellitus in the UK Biobank

**DOI:** 10.1136/bmjdrc-2024-004826

**Published:** 2025-09-08

**Authors:** Dale Handley, Alexandra C Gillett, Renu Bala, Jessica Tyrrell, Cathryn M Lewis

**Affiliations:** 1 NIHR Maudsley Biomedical Research Centre, South London and Maudsley NHS Foundation Trust, London, UK; 2 Clinical and Biomedical Science, Institute of Health and Life Sciences, University of Exeter, Exeter, UK

**Keywords:** Depression, HbA1c, Classification, Diabetes Mellitus, Type 2

## Abstract

**Introduction:**

Frequent glycated hemoglobin A1c (HbA1c) monitoring is recommended in individuals with type 2 diabetes mellitus (T2D). We aimed to identify distinct, long-term HbA1c trajectories following a T2D diagnosis and investigate how these glycemic control trajectories were associated with health-related traits and T2D complications.

**Research design and methods:**

A cohort of 12,435 unrelated individuals of European ancestry with T2D was extracted from the UK Biobank data linked to primary care records. Latent class growth mixture modeling was applied to identify classes with similar HbA1c trajectories over the 10 years following T2D diagnosis. Associations between HbA1c class membership and sociodemographic factors, biomarkers, polygenic scores, and T2D-related outcomes, were tested using logistic regression and Cox proportional hazards models.

**Results:**

Six HbA1c trajectory classes were identified. The largest class (76.8%) maintained low and stable HbA1c levels over time. Five additional smaller classes with distinct, but more variable, trajectories were found and were associated with younger age at T2D diagnosis, higher fasting glucose levels, higher random glucose levels, higher body mass index polygenic score and increased healthcare use before T2D diagnosis. Relative to the low and stable class, these five showed increased risks of T2D complications, including stroke (HR=1.55 (1.31–1.84)), kidney disease (HR=1.39 (1.27–1.53)), all-cause mortality (HR=1.36 (1.23–1.51)), and progression to combination therapy (HR=3.22 (3.04–3.41)) or insulin (HR=3.21 (2.89–3.55)).

**Conclusion:**

Individuals with T2D who show higher and more variable HbA1c trajectories are at increased risk of developing T2D-related complications. Early identification of patients at risk, based on factors such as age at diagnosis and previous healthcare utilization could improve patient outcomes.

**WHAT IS ALREADY KNOWN ON THIS TOPIC**

- Type 2 diabetes (T2D) is a highly heterogeneous disease.
- Higher and more variable glycated hemoglobin A1c (HbA1c) during T2D disease course is strongly associated with risk of vascular complications and mortality.

**WHAT THIS STUDY ADDS**

- This study identifies six distinct HbA1c trajectories following T2D diagnosis. Having an atypical HbA1c trajectory is associated with increased risk of rapid disease progression, vascular complications and all-cause mortality.
- The body mass index polygenic score, healthcare use prior to diagnosis, and age at T2D onset aid in predicting which individuals will have an atypical trajectory.

**HOW THIS STUDY MIGHT AFFECT RESEARCH, PRACTICE OR POLICY**

- The incorporation of polygenic scores and healthcare use prior to diagnosis into clinical prediction models might help to identify individuals that would benefit from increased HbA1c monitoring and early and intensive antiglycemic intervention.

## Introduction

 Type 2 diabetes mellitus (T2D) is a chronic metabolic disorder characterized by persistent hyperglycemia due to insulin resistance and impaired insulin secretion.^1^ T2D currently affects approximately 6.1% of the global population, and is projected to affect over 1.3 billion individuals globally by 2050 without effective prevention.^2^

The pathophysiological stress of T2D extends to nearly all organ systems, leading to severe complications, which are usually categorized as microvascular or macrovascular. Microvascular complications, primarily nephropathy, neuropathy, and retinopathy, result from damage to small blood vessels.^3^ In contrast, macrovascular complications stem from damage to large vessels and are linked to accelerated atherosclerosis, leading to a high incidence of coronary artery disease, cerebrovascular disease, and peripheral artery disease among T2D patients.^4^ Additionally, major depressive disorder (MDD) is a notable comorbidity; individuals with T2D are at greater risk of MDD compared with normoglycemic populations, and a pre-existing diagnosis of MDD increases the risk of developing T2D by 32%.^5^ Incident MDD following T2D leads to suboptimal glycemic control, increasing the risk of vascular complications and all-cause mortality.^6 7^

Optimal management of T2D, including maintaining effective glycemic control, is important for improving health outcomes and patient quality of life.^8^ Early and intensive glycemic control after T2D diagnosis is essential for reducing mortality risk, while later intervention confers only modest improvements.^9^ In clinical practice, glycemic control is typically assessed using glycated hemoglobin A1c (HbA1c), with measurements required every three to 6 months for effective monitoring. Elevated and highly variable HbA1c measures are robust predictors of complications, suggesting that understanding the different patterns of HbA1c over years since T2D diagnosis can inform more personalized management strategies.^10^

Latent class growth mixture modeling (LCGMM) is an extension of linear mixed modeling that identifies distinct subpopulations within longitudinal data which can reveal shared disease risk.^11^ To date, a limited number of studies have applied LCGMM to identify HbA1c trajectories over time since T2D diagnosis using routinely collected clinical data. A recent study demonstrated that non-stable trajectories are associated with increased risk of microvascular complications and mortality, while another showed that body mass index (BMI), HbA1c and triglycerides measured at T2D diagnosis were the most important predictors of trajectory class membership.^12 13^ However, these studies focused on either the association with complications, or on identifying predictors of trajectory membership, but did not consider both comprehensively. Additionally, no existing studies have investigated associations between identified classes and healthcare utilization, MDD or genetic predictors.

Our study leverages the extensive UK Biobank (UKB) study linked to primary care records to identify subgroups of T2D by HbA1c trajectories. We applied LCGMM to identify HbA1c trajectories over a 10-year period following T2D diagnosis and identified exposures associated with class membership, considering variables measured at or prior to T2D diagnosis, including polygenic scores and MDD diagnosis. Furthermore, we examined the associations between these trajectories and T2D complications, all-cause mortality and medications, such as progression to insulin therapy. By incorporating a diverse range of class predictors and health outcomes in a large dataset, we aim to provide a comprehensive approach to understanding glycemic control in T2D.

## Methods

### Data

The UKB is a prospective health study of ∼500,000 individuals recruited between the ages of 40 and 70 between 2006 and 2010.^14^ UKB collects a wide range of data including physical measurements (such as HbA1c), detailed health questionnaires and genetic data. Linkage to general practice (GP) primary care data is available for 46% of UKB participants, providing diagnostic codes, clinical biochemistry tests, clinical events, and prescribing data between the years 1990 and 2017 (online supplemental figure 1).

### Study population

The study population consisted of UKB participants with T2D diagnosis and linked primary care data available. Participants with T2D were identified via a previously validated approach, using both primary care and UKB-collected data.^15^ Briefly, individuals must meet at least two of the following criteria: any HbA1c measurement >48 mmol/mol, any prescription for glucose-lowering medication, any self-report for T2D, a hospital episode statistics (HES) International Classification of Diseases 9/10 code for T2D, or a GP-based T2D diagnosis. The index date was T2D diagnosis date, defined as the earliest occurrence of any of these criteria. To avoid the inclusion of misdiagnosed non-T2D diabetes cases, we excluded individuals aged <35 years at T2D diagnosis, or those prescribed insulin within a year of diagnosis. Several additional exclusion criteria were included: (1) no recorded HbA1c measurement >38 mmol/mol within 6 months of diagnosis, (2) fewer than three HbA1c measurements available within 10 years of diagnosis, (3) no available genotype data, and (4) an undated MDD diagnosis.

This study uses polygenic scores created using summary statistics from European ancestry genome-wide association studies. Therefore, we further restricted our analyses to unrelated individuals of European ancestry (online supplemental information, methods S1).

### HbA1c measurements

HbA1c values were extracted from the UKB assessments and primary care records (online supplemental table 1). Further information for HbA1c quality control is presented in online supplemental information, methods S2. Longitudinal HbA1c measurements starting from T2D diagnosis were used as the outcome when identifying subgroups of glycemic control trajectories, with a maximum follow-up of 10 years (online supplemental information, methods S3).

### Statistical Analysis

#### Identifying HbA1C trajectories

LCGMM was used to identify classes of individuals with similar HbA1C trajectories over the first decade following their T2D diagnosis. In LCGMM, convergence issues can arise when very short time intervals between observations are present. To avoid this, only one HbA1c measurement per individual every 0.1 years was included. For individuals with two measures within 0.1 years (<2% of individuals), the mean value was used. A restricted cubic spline function with three knot points was used to capture non-linear trends in HbA1c over time in the fixed effects models. A full description of the model selection criteria is provided in online supplemental methods, S3. Class characteristics at the index date were summarized. Between-class differences were assessed using Kruskal-Wallis tests for continuous variables and likelihood ratio tests for binary variables, by comparing logistic regression models with and without the LCGMM class variable.

#### Testing for association of exposures with HbA1c trajectories

Two complementary approaches were used to examine the associations between exposures, measured at or prior to T2D diagnosis, and class membership. First, we applied multinomial logistic regression (MLR) to identify exposures associated with each class relative to the reference class (the most common class). Second, we used logistic regression to the most common class (the reference) versus all other identified classes combined. This allowed us to identify exposures associated with deviating from the most common HbA1c trajectory. All models were adjusted for genetic sex and age at T2D diagnosis. Holm-Bonferroni correction was applied to account for multiple testing, with adjusted p values presented. Therefore, p<0.05 was considered statistically significant for all analyses.

##### Exposures

A range of demographic, biomarker, pre-existing diagnoses, polygenic scores, and healthcare utilization variables were considered. Demographic exposures considered were genetic sex, number of education years, smoking status (ever vs never) and Townsend deprivation index (TDI), as defined by UKB fields (online supplemental table 1). Age at T2D diagnosis was defined as the difference between a participant’s T2D diagnosis date and date of birth. As the day of birth is unavailable in the UKB, this was set to the first of the month. The following biomarker variables were included, created following previous definitions^16^ and using primary care and UKB assessment data: BMI, fasting blood glucose, random blood glucose, diastolic blood pressure, systolic blood pressure, total blood cholesterol, high-density lipoprotein, low-density lipoprotein, and triglycerides.^16^ For all biomarkers, only measurements taken up to 1 month before T2D diagnosis were considered. Healthcare utilization exposures were the number of GP visits, the number of unique HES dates and the number of unique prescription dates. The number of GP visits was calculated using the number of unique dates for which an individual had at least one primary care code for both 1 month and 1 year prior to T2D diagnosis. The total number of unique hospital dates, and total number of unique prescription dates were calculated similarly. Pre-existing diagnoses considered were stroke, cardiovascular disease (CVD), and MDD, defined using previously described methods based solely on primary care records.^16 17^ Polygenic scores for T2D, BMI, and MDD were generated and validated in UKB using Polygenic risk score - continuous shrinkage (PRS-CS) software via the GenoPred pipeline (online supplemental information, methods S4, S5 and Result S1).^18 19^

### Testing for association of HbA1c trajectories with secondary outcomes

Time-to-event analysis was performed using Cox proportional hazards modeling to determine whether class membership increased the risk of developing secondary outcomes. The time of entry was defined as the T2D diagnosis date. When the secondary outcome was all-cause mortality, the censoring date was the last date the national death registry linkage was updated (November 22, 2021). For all other outcomes, the censoring date was the earlier of the date of death or the last date of data collection. All models were adjusted for age at T2D diagnosis, genetic sex, years of education, TDI, and ever-smoked status. The reference group for class membership was the modal class. All p values are reported as Holm-Bonferroni corrected values. Proportional hazard assumption was evaluated for all time-to-event models using scaled Schoenfeld residuals and the Grambsch-Therneau test.^20^ Where violations were identified, we conducted sensitivity analyses using Royston-Parmar proportional odds models, which do not rely on the proportional hazards assumption (online supplemental information, methods S6). Non-proportional hazards were not modeled directly using time-by-class interactions due to the limited sample sizes in some latent class groups.

#### Secondary outcomes

The following eight phenotypes were used as outcomes in the time-to-event analyses: all-cause mortality, all CVD combined, diabetic kidney disease, all strokes combined, peripheral artery disease, MDD, progression to combination T2D therapy, and progression to insulin. All-cause mortality was defined using the linked Office for National Statistics death registry. Peripheral artery disease was defined and MDD according to previous work.^17 21^ All other outcomes were obtained using previously defined UKB inclusion and exclusion code lists and fields for T2D complications.^16^ Additionally, two medication phenotypes, “progression to insulin” and “progression to combination therapy” were included as measures of T2D disease progression rate (online supplemental information, methods S7).

### Software and code availability

All analyses were performed using R V.4.2.0. LCGMM and model selection used the *lcmm* and *LCTMToolkit* packages.^22 23^ MLR was conducted using the *nnet* package.^24^ The *survival* package was used for Cox proportional hazards modeling.^25^ Figures and tables were created using *ggplot2* and *gt.*^26 27^ Code for this project is available at https://github.com/dale-handley/UKBIOBANK-HbA1c-LCGMM.

### Reporting guidelines

This study adheres to the STROBE (*Strengthening the Reporting of Observational Studies in Epidemiology*) guidelines for observational research. A completed checklist is appended to the online supplemental information.^28^

### Data availability

The data that support the findings of this study are available from UKB, but restrictions apply to the availability of these data, which were used under license for the current study and therefore are not publicly available.

### Results

#### Cohort characteristics

In total, 12,435 individuals met the study inclusion criteria (online supplemental figure 2). The cohort was predominantly male (61.6%), with an average age at T2D diagnosis of 59.7 years (table 1, online supplemental table 2). The median follow-up time was 8 years, with a median of 13 HbA1c measurements available.

**Table 1:** Descriptive statistics at T2D diagnosis date for all individuals, stratified by assigned LCGMM class

	Class A Low and stable N=9549	Class B Low parabolic N=1126	Class C High parabolic N=245	Class D Steep increase N=315	Class E Slow decrease N=326	Class F Rapid decrease N=874	P value
Demographic information							
Sex (male)	,5784 (61%)	727 (65%)	144 (59%)	192 (61%)	232 (71%)	575 (66%)	3.3×10–5
Age (years)	60.3 (54.7 to 65.2)	56.1 (50.7 to 61.8)	54.0 (47.6 to 60.1)	51.9 (46.5 to 59.2)	54.5 (49.3 to 60.3)	58.9 (52.4 to 63.6)	6.0×10–120
Ever smoked	6,381 (67%)	754 (67%)	165 (67%)	212 (68%)	209 (65%)	583 (67%)	1.0×10–20
Education (years)	10 (7.0 to 15.0)	10.0 (7.0 to 15.0)	10.0 (7.0 to 15.0)	10.0 (7.0 to 15.8)	10.0 (7.0 to 15.0)	11.0 (7.0 to 16.0)	0.61
TDI	−1.7 (−3.4 to 1.3)	−1.4 (−3.2 to 1.6)	0.0 (-2.3 to 3.0)	−0.1 (−2.8 to 3.0)	−1.1 (−3.1 to 2.2)	−1.3 (−3.1 to 2.0)	7.5×10–15
Cardiometabolic biomarkers							
HbA1c (mg/dL)	50.6 (46.0 to 56.0)	54.3 (49.1 to 64.0)	59.9 (50.8 to 80.0)	56.0 (50.0 to 73.0)	91.4 (77.2 to 103.8)	92.3 (78.0 to 109.0)	<1×10-200
Fasting glucose (mmol/mol)	7.4 (6.9 to 8.1)	7.9 (7.3 to 9.0)	8.7 (7.4 to 11.3)	7.7 (7.2 to 9.2)	11.9 (9.8 to 15.0)	14.0 (11.0 to 17.1)	5.1×10–53
Random glucose (mmol/mol)	8.9 (7.4 to 11.7)	9.1 (7.4 to 11.6)	9.6 (7.5 to 12.3)	9.6 (7.9 to 16.0)	15.3 (13.3 to 22.1)	16.6 (13.0 to 19.8)	1.3×10–51
HDL (mmol/mol)	1.2 (1.0 to 1.4)	1.1 (1.0 to 1.3)	1.0 (0.9 to 1.2)	1.0 (0.9 to 1.3)	1.1 (1.0 to 1.3)	1.1 (1.0 to 1.3)	0.019
LDL (mmol/mol)	3.0 (2.3 to 3.7)	3.2 (2.1 to 3.9)	3.1 (2.5 to 3.8)	3.5 (2.6 to 4.0)	3.3 (2.7 to 3.8)	3.2 (2.5 to 3.9)	0.31
Total cholesterol (mmol/mol)	5.2 (4.3 to 6.0)	5.2 (4.4 to 6.3)	5.1 (4.3 to 5.5)	5.6 (5.0 to 6.6)	5.9 (4.9 to 6.3)	5.5 (4.7 to 6.6)	1.2×10–5
Triglycerides (mmol/mol)	2.0 (1.5 to 2.8)	2.2 (1.6 to 2.9)	2.3 (1.4 to 3.4)	2.5 (1.8 to 3.2)	2.6 (1.8 to 4.1)	2.2 (1.6 to 2.7)	4.6×10–4
Anthropometric traits							
BMI (kg/m2)	32.0 (28.8 to 36.0)	33.1 (30.0 to 37.6)	3.53 (3.01 to 3.93)	34.3 (28.6 to 37.6)	33.2 (28.7 to 36.1)	30.7 (27.2 to 35.6)	5×10–3
Systolic blood pressure (mm Hg)	140 (130 to 153.0)	140 (132.0 to 153.0)	140.0 (129.5 to 150.3)	146.0 (137.0 to 160.0)	142.0 (133.5 to 156.3)	140.0 (130.0 to 160.0)	0.15
Diastolic blood pressure (mm Hg)	82.0 (78.0 to 90.0)	85.0 (80.0 to 93.0)	80 (75.8 to 90.3)	85.0 (80.0 to 90.0)	85.5 (80.0 to 95.3)	84.0 (80.0 to 92.0)	1×10–3
Pre-existing comorbidities							
MDD	884 (9.3%)	97 (8.6%)	22 (9.0%)	34 (11.0%)	27 (8.3%)	61 (7.0%)	0.22
CVD	1,026 (11%)	122 (11%)	30 (12%)	28 (8.9%)	25 (7.7%)	65 (7.4%)	0.01
Stroke	332 (3.4%)	31 (3.3%)	8 (3.3%)	8 (2.5%)	9 (2.8%)	20 (2.3%)	0.42
Inclusion criteria							
HbA1c over 48 mmol/mol	8,751 (92%)	1,126 (100%)	245 (100%)	315 (100%)	325 (100%)	874 (100%)	2.2×10–92
Prescribed T2D medication	6,803 (97%)	1,097 (99%)	234 (98%)	310 (99%)	324 (100%)	848 (100%)	2.5×10–9
Primary care T2D code	8,117 (85%)	917 (81%)	207 (84%)	259 (82%)	261 (80%)	713 (82%)	1×10–3
HES T2D code	7,233 (76%)	911 (81%)	197 (80%)	254 (81%)	248 (76%)	638 (73%)	7.5×10–5
Self-reported T2D diagnosis	4,122 (49%)	600 (65%)	93 (48%)	173 (73%)	154 (58%)	394 (52%)	2.2×10–28
Healthcare utilization							
HES dates (1mo)	0.0 (0.0 to 1.0)	0.0 (0.0 to 1.0)	0.0 (0.0 to 1.0)	0.0 (0.0 to 1.0)	0.0 (0.0 to 1.0)	0.0 (0.0 to 1.0)	0.04
HES dates (1yr)	1.0 (0.0 to 2.0)	1.0 (0.0 to 2.0)	1.0 (0.0 to 2.0)	1.0 (0.0 to 2.0)	1.0 (0.0 to 3.0)	1.0 (0.0 to 2.0)	2.9×10–5
Prescription dates (1mo)	0.0 (0.0 to 1.0)	0.0 (0.0 to 0.0)	0.0 (0.0 to 0.0)	0.0 (0.0 to 0.0)	0.0 (0.0 to 0.0)	0.0 (0.0 to 1.0)	1.1×10–8
Prescription dates (1yr)	3.0 (0.0 to 6.0)	2.0 (0.0 to 4.0)	1.0 (0.0 to 5.0)	1.0 (0.0 to 4.0)	1.0 (0.0 to 4.0)	2.0 (0.0 to 5.0)	1.4×10–33
Primary care dates (1mo)	2.0 (1.0 to 4.0)	2.0 (1.0 to 3.0)	2.0 (1.0 to 3.0)	2.0 (1.0 to 3.0)	2.0 (1.0 to 3.0)	2.0 (1.0 to 3.0)	4.7×10–18
Primary care dates (1yr)	9.0 (5.0 to 15.0)	7.0 (3.0 to 13.0)	6.0 (2.0 to 13.0)	6.0 (2.0 to 12.0)	5.0 (2.0 to 10.0)	7.0 (4.0 to 12.0)	1.2×10–49

BMI, body mass index; CVD, cardiovascular disease; HbA1c, glycated hemoglobin A1c; HDL, high-density lipoprotein; HES, hospital episode statistics; LCGMM, latent class growth mixture modeling; LDL, low-density lipoprotein; MDD, major depressive disorder; 1mo, only values within 1 month prior to diagnosis included; T2D, type 2 diabetes; TDI, Townsend deprivation index; 1yr, only values with 1 year prior to diagnosis were included.

#### Latent class growth mixture modeling

**Figure 1:**
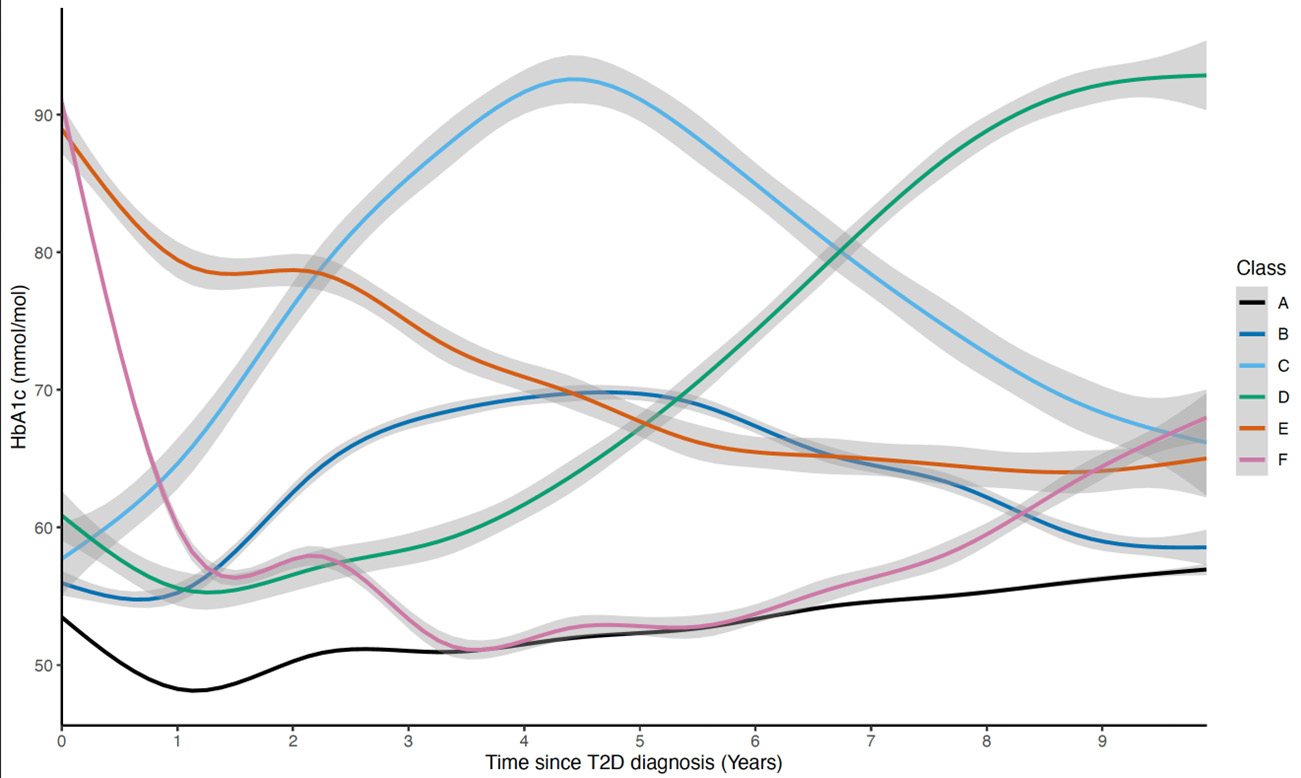
Mean HbA1c trajectories during the first 10 years post-T2D diagnosis for all classes. Shaded area represents 95% CIs. HbA1c, glycated hemoglobin A1c; T2D, type 2 diabetes.

In the analysis of 10-year HbA1c trajectories among individuals newly diagnosed with T2D, six models converged within the designated time frame. The model with six classes was the best fitting, meeting the predefined selection criteria (onlinesupplemental table 3  4). Most individuals were assigned to Class A (76.8%), which had the lowest initial HbA1c of any class, which remained low and slowly increased over time (“low and stable”). This class is the reference class in subsequent analyses. The other five classes had distinct trajectories that were typically higher and more variable: (1) Class B (9.06%) showed low HbA1c levels at diagnosis that moderately increased until year 5, then gradually returned to initial levels (“low parabolic”), (2) Class C (1.97%) started low, rapidly increased until 4.5 years after diagnosis (“high parabolic”), (3) Class D (2.53%) had a steep increase in HbA1c after 2 years (“steep increase”), (4) Class E (2.62%) started high and slowly decreased during the 10 years following T2D diagnosis (“slow decrease”) and (5) Class F (7.03%) had high HbA1c levels at diagnosis that rapidly decreased over the first 18 months, then gradually increased from the 4th year (“rapid decrease”) (figure 1 and online supplemental figure 3).

#### Exposures associated with class membership

Compared with Class A (“low and stable”), membership of all other classes (B–F) was associated with a younger age at T2D diagnosis (table 2). Additionally, higher fasting glucose levels at T2D diagnosis were associated with increased odds of class membership relative to Class A, in all classes except Class D (“steep increase”). Higher TDI was linked with increased odds of belonging to Classes C (“high parabolic”; OR=1.09, 95% CI (1.05 to 1.13)) and D (OR=1.10 (1.04 to 1.11)), while higher random glucose levels at T2D diagnosis increased the odds of membership to Class E (“slow decrease”; OR=1.36 (1.29 to 1.45)) and Class F (“rapid decrease”; OR=1.34 (1.28 to 1.39)) relative to Class A. Males were more likely than females to belong to Class E compared with Class A (OR=1.60 (1.25 to 2.04)). Finally, membership of Class F was associated with higher levels of total cholesterol (OR=1.34 (1.19 to 1.52)), triglycerides (OR=1.18 (1.09 to 1.28)) and T2D polygenic score (OR=1.15 (1.07 to 1.24)).

**Table 2:** Associations between exposures and HbA1c class membership, adjusted for sex and age at T2D diagnosis

	Class B Low parabolic N=1126		Class C High parabolic N=245		Class D Steep increase N=315		Class E Slow decrease N=326		Class F Rapid decrease N=874		Classes B–F combined	
Demographic information												
Demographic information	OR [95% CI]	P value	OR [95% CI]	P value	OR [95% CI]	P value	OR [95% CI]	P value	OR [95% CI]	P value	OR [95% CI]	P value
Sex (male)	1.18 [1.04 to 1.34]	1	0.92 [0.71 to 1.20]	1	1.01 [0.80 to 1.27]	1	1.60 [1.25 to 2.04]	0.022	1.25 [1.08 to 1.44]	0.401	1.19 [1.09 to 1.30]	**0.01**
Age (years)	0.94 [0.94 to 0.95]	**9E–48**	0.92 [0.91 to 0.94]	**4.42×10–24**	0.90 [0.89 to 0.91]	**5.3×10–50**	0.93 [0.92 to 0.94]	**2.82×10–27**	0.98 [0.97 to 0.98]	**9.5×10–5**	0.94 [0.94 to 0.95]	**8.6×10–97**
Ever smoked	1.06 [0.93 to 1.21]	1	1.13 [0.86 to 1.48]	1	1.17 [0.92 to 1.50]	1	0.93 [0.73 to 1.18]	1	0.99 [0.85 to 1.15]	1	1.04 [0.95 to 1.14]	1
Education (years)	0.98 [0.97 to 0.99]	**0.192**	0.97 [0.94 to 0.99]	1	0.98 [0.95 to 1]	1	0.96 [0.94 to 0.99]	0.452	1.00 [0.99 to 1.02]	1	0.98 [0.97 to 0.99]	**0.028**
TDI	1.01 [0.99 to 1.03]	1	1.09 [1.05 to 1.13]	**0.0003**	1.08 [1.04 to 1.11]	**0.002**	1.03 [1.00 to 1.06]	1	1.03 [1.01 to 1.05]	1	1.03 [1.02 to 1.05]	**1.3×10–13**
Cardiometabolic biomarkers												
Fasting glucose (mmol/mol)	1.22 [1.13 to 1.32]	**3.9×10–5**	1.38 [1.20 to 1.58]	**9.8×10–4**	1.15 [0.98 to 1.35]	1	1.63 [1.46 to 1.82]	**3.7×10–16**	1.80 [1.67 to 1.95]	**8.9×10–47**	1.43 [1.36 to 1.51]	**1.4×10–40**
Random glucose (mmol/mol)	0.99 [0.94 to 1.04]	1	1.03 [0.94 to 1.14]	1	1.12 [1.03 to 1.21]	1	1.36 [1.29 to 1.45]	**1.5×10–23**	1.34 [1.28 to 1.39]	**5.7×10–40**	1.16 [1.13 to 1.20]	**5.6×10–27**
Inclusion criteria												
Primary care T2D code	1.89 [1.20 to 2.98]	0.806	1.71 [0.74 to 3.93]	1	1.17 [0.64 to 2.13]	1	0.67 [0.40 to 1.11]	1	2.00 [1.14 to 3.50]	1	1.43 [1.09 to 1.90]	1
HES T2D code	1.41 [1.20 to 1.65]	**0.003**	1.40 [1.01 to 1.93]	1	1.46 [1.09 to 1.94]	1	1.08 [0.83 to 1.40]	1	0.87 [0.75 to 1.02]	1	1.17 [1.05 to 1.29]	**0.405**
Self-reported T2D diagnosis	0.76 [0.64 to 0.90]	0.184	0.66 [0.47 to 0.90]	1	0.61 [0.46 to 0.80]	0.061	0.72 [0.54 to 0.97]	1	0.86 [0.70 to 1.05]	1	0.74 [0.66 to 0.84]	**1.7×10–4**
Healthcare utilization												
HES dates (1 month)	0.95 [0.86 to 1.06]	1	1.09 [0.88 to 1.34]	1	1.17 [0.98 to 1.40]	1	1.04 [0.86 to 1.25]	1	0.92 [0.81 to 1.04]	1	0.99 [0.92 to 1.06]	1
HES dates (1 year)	0.98 [0.94 to 1.02]	1	1.00 [0.92 to 1.08]	1	1.11 [1.04 to 1.19]	0.148	0.95 [0.88 to 1.02]	1	0.91 [0.87 to 0.96]	**0.041**	0.97 [0.95 to 1]	1
Prescription dates (1 month)	0.85 [0.76 to 0.95]	0.407	0.82 [0.65 to 1.04]	1	0.91 [0.75 to 1.11]	1	0.90 [0.74 to 1.10]	1	1.08 [0.97 to 1.20]	1	0.94 [0.87 to 1]	1
Prescription dates (1 year)	0.96 [0.94 to 0.98]	**0.008**	0.97 [0.94 to 1.01]	1	0.98 [0.95 to 1.02]	1	0.96 [0.92 to 0.99]	1	0.99 [0.97 to 1.01]	1	0.97 [0.96 to 0.98]	**0.003**
Primary care dates (1 month)	0.95 [0.92 to 0.98]	0.398	0.93 [0.86 to 1.00]	1	0.98 [0.92 to 1.04]	1	0.87 [0.81 to 0.93]	**0.008**	0.98 [0.94 to 1.01]	1	0.95 [0.93 to 0.97]	**0.005**
Primary care dates (1 year)	0.99 [0.98 to 0.99]	0.098	0.99 [0.98 to 1.01]	1	0.99 [0.98 to 1.01]	1	0.97 [0.95 to 0.98]	**0.014**	0.98 [0.97 to 0.99]	**2.2×10–4**	0.98 [0.98 to 0.99]	**2.2×10–6**
Polygenic scores												
BMI PGS	1.11 [1.04 to 1.19]	0.13	1.07 [0.94 to 1.22]	1	1.19 [1.06 to 1.34]	0.405	1.10 [0.98 to 1.24]	1	1.04 [0.97 to 1.12]	1	1.09 [1.05 to 1.14]	**0.009**
T2D PGS	1.06 [0.99 to 1.13]	1	0.98 [0.86 to 1.12]	1	1.04 [0.92 to 1.17]	1	1.02 [0.90 to 1.14]	1	1.15 [1.07 to 1.24]	**0.034**	1.07 [1.02 to 1.12]	0.405
MDD PGS	0.98 [0.93 to 1.05]	1	0.99 [0.87 to 1.12]	1	1.11 [0.99 to 1.24]	1	1.02 [0.91 to 1.14]	1	0.96 [0.90 to 1.03]	1	0.99 [0.95 to 1.04]	1

The effect size is reported as OR and 95% CIs in square brackets for each class, compared with the reference class (A, “low and stable”). To generate the overall non-reference class models (B–F), all individuals who were not assigned to class A were considered a single class.

Values in bold represent Holm-Bonferroni adjusted p values which are statistically significant (p<0.05).

BMI, body mass index; HbA1c, glycated hemoglobin A1c; HES, hospital episode statistics; MDD, major depressive disorder; PGS, polygenic scores; T2D, type 2 diabetes; TDI, Townsend deprivation index.

Comparing individuals in reference Class A with all other classes combined showed that membership of classes B–F was associated with a younger age at T2D diagnosis (OR=0.95 (0.94 to 0.95)), being male (OR=1.19 (1.09 to 1.30)), having fewer years in education (OR=0.98 (0.97 to 0.99)) and higher levels of TDI (OR=1.03 (1.02 to 1.05)), fasting glucose (OR=1.43 (1.36 to 1.51)), random glucose (OR=1.16 (1.13 to 1.20)) and BMI polygenic score (OR=1.09 (1.05 to 1.14)).

#### Source of T2D diagnosis criteria and healthcare utilization is associated with class membership

Given the high HbA1c intercept values observed in Classes E and F, we examined the association between class membership and healthcare utilization, as well as the source of T2D diagnosis, to assess whether diagnosis in some classes may have occurred secondary to another clinical investigation or during emergency care (table 2 and online supplemental table 5). Compared with Class A, individuals in the non-reference class were less likely to self-report a diagnosis of T2D (OR=0.74 (0.66 to 0.84)). Fewer hospital episode dates in the year prior to T2D diagnosis were also associated with membership of Class F (OR=0.91 (0.87 to 0.96)). Additionally, having fewer primary care dates within the year before diagnosis was associated with higher odds of belonging to Class E (OR=0.97 (0.95 to 0.98)) and Class F (OR=0.98 (0.97 to 0.99)) compared with Class A. Additionally, belonging to a non-reference class was associated with fewer GP visits in both the year (OR=0.98 (0.98 to 0.99)) and month (OR=0.95 (0.93 to 0.97)) prior to T2D diagnosis, and with fewer prescriptions in the year before diagnosis (OR=0.97 (0.96 to 0.99)), indicating that reduced healthcare utilization is associated with non-reference class membership.

#### Class membership associations with secondary outcomes

**Figure 2:**
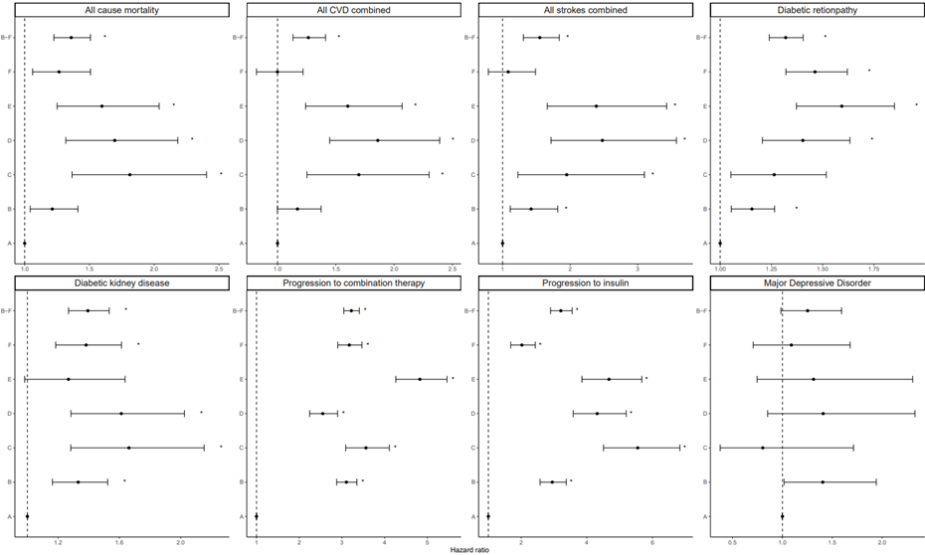
Association of class membership with T2D outcomes, using time-to-event analysis. Error bars represent 95% CIs. Asterisks represent statistically significant p values after applying. CVD, cardiovascular disease; T2D, type 2 diabetes.

Secondary outcomes were strongly associated with class membership after correcting for multiple testing (figure 2; onlinesupplemental table 6  7). All non-reference classes showed an increased risk of developing diabetic retinopathy relative to Class A. Class E (“slow decrease”) had the highest risk (1.43 95% CI: 1.26 to 1.61) compared with those in Class A, while Class B (“low parabolic”) had the lowest relative risk (HR=1.25 (1.13 to 1.38)). Higher rates of all-cause mortality, stroke and CVD events were seen in classes C (“high parabolic”), D (“steep increase”) and E compared with Class A. For example, for stroke, the hazard rate ranged from 2.07 (95% CI: 1.28 to 3.34) for Class C, to 2.72 (95% CI: 1.88 to 3.94) for Class D, compared with class A. All classes except Class E were at increased risk of developing kidney disease compared with Class A.

All classes were associated with progression to dual therapy and to insulin. Class F (“rapid decrease”) had the lowest estimated HR for both medication outcomes, being 1.77 (95% CI: 1.45 to 2.16) times more likely to progress to combination therapy and 1.87 (95% CI: 1.63 to 2.14) times more likely to progress to insulin compared with Class A. Class E had the highest risk of progressing to combination therapy (HR=4.04 (3.38 to 4.81)) and Class C had the highest risk of progressing to insulin (HR=5.67 (4.59 to 7.02)). There was no difference in the risk of MDD diagnosis for any class compared with the reference.

When Classes B–F were combined into one group (atypical trajectories) and compared with Class A (typical), we observed that belonging to an atypical trajectory (Classes B–F) was associated with an increased risk of experiencing all secondary outcomes considered, except for MDD.

For the time-to-event sensitivity analyses, we found that 8 out of 15 class—outcome models contained at least one exposure which violated the proportional hazards assumption (online supplemental table 8). Using the Royston-Palmer models, we found that all associations that were identified using the Cox proportional hazards models remained statistically significant after applying the Royston-Palmer models, except for the association of Class B with diabetic retinopathy (OR=1.14 (1.01 to 1.29), p=0.11) (online supplemental table 9). Therefore, it is likely that the conclusions drawn from using the Cox models are valid, even in the presence of proportional hazards violations.

## Discussion

In this study, we used the extensive data available in the UKB and identified six classes of individuals of European ancestry with T2D based on their HbA1c trajectories over a mean follow-up of 8.1 years from diagnosis. The reference class (A; “low and stable”) represents a trajectory previously observed in other studies. This class has the lowest initial HbA1c levels of all classes and experiences a dip around 1 year after diagnosis, before rebounding and slowly increasing thereafter.^13 15^ Classes B (“low parabolic”) and C (“high parabolic”) represent individuals who experience increases in HbA1c during the first 5 years post-diagnosis, followed by a decline, with Class C experiencing a more pronounced increase and subsequent decline than Class B. Classes E (“slow decrease”) and F (“rapid decrease”) contain individuals with high HbA1c levels at diagnosis, that decrease over time, with Class F having a higher initial HbA1c and a steeper decline. Class D (“steep increase”) has a moderately high initial HbA1c, with a rapid increase approximately 4 years postdiagnosis. Overall, 23.2% of participants were members of Classes B–F (non-reference/atypical classes), characterized by higher and more variable HbA1c trajectories compared with the low and stable trend observed in Class A (reference/typical class).

Younger age of T2D onset and higher fasting blood glucose at T2D diagnosis were associated with class membership for nearly all classes, compared with Class A. While the association with fasting glucose is expected due to its strong correlation with HbA1c, the link between younger age of T2D onset and class membership indicates that individuals who develop T2D at a younger age tend to have poorer glycemic control and a higher risk of developing complications. Possible explanations for this include having higher adiposity, showing lower adherence to T2D medications, and/or having a genetically distinct phenotype which is associated with different risks of T2D complications.^29 30^ This finding aligns with previous studies, which have identified younger age of T2D onset as a risk factor for developing vascular complications.^31^

In the all-classes combined analysis, higher BMI polygenic score was associated with non-reference class membership, but phenotypically measured BMI was not. This could suggest a pleiotropic genetic effect of the BMI polygenic score on body fat composition, which is strongly associated with the development of T2D and complications.^32 33^ Additionally, the BMI polygenic score may be capturing changes in BMI that occur alongside the HbA1c trajectories which may not be captured by a single phenotypic BMI measurement.^34^

In the healthcare utilization analysis, we found a strong link between belonging to a non-reference class and reduced GP visits before T2D diagnosis. Since increased GP visitation is associated with lower hospitalization rates in T2D patients and early diagnosis is crucial for glycemia management, these results suggest that lower healthcare utilization prior to diagnosis could lead to a more advanced T2D at diagnosis, poorer glycemic control, and higher risk of postdiagnosis complications.^35^

The time-to-event analysis of secondary outcomes showed that all non-standard classes were at increased risk of developing all disease outcomes, except MDD, compared with Class A. While the association between atypical HbA1c trajectories and risk of T2D complications is well established, we identified three classes (C, D, E) at notably elevated risk of diabetes-related complications and all-cause mortality. The observed differences between Classes E and F (slow vs rapid decrease) demonstrate total exposure to high HbA1c levels and early glycemic management as important risk factors for the development of complications, as reported previously.^36 37^ Similarly, the increased HbA1c variability when comparing Classes B and C (low vs high parabolic) underscores the need for longer-term and more intensive HbA1c management on the observation of rapidly increasing HbA1c. Class D may reflect poor response to second-line T2D therapies or therapeutic inertia, where delayed treatment adjustments lead to worse long-term glycemic control and higher complication risk.^38^

This UKB study of T2D HbA1c trajectories offers several advantages over previous research. First, the strict T2D definition increases the likelihood of including only true T2D cases. Second, the large sample sizes, stringent model selection, and flexible time modeling ensure that LCGMM-generated trajectories represent meaningful HbA1c subclasses.^39^ Third, we consider a broad range of exposures and secondary outcomes, allowing a comprehensive investigation of how these trajectories develop and affect the clinical course of T2D in the UKB. Finally, this is the first study to incorporate genetic information into LCGMM, revealing a novel association between the BMI polygenic score, but not BMI, and atypical HbA1c trajectories, alongside a class-specific association between the T2D polygenic score and Class F.

However, our study has several limitations. First, LCGMM is computationally intensive. It is therefore possible that with more computational power, a model with more classes could have been selected. However, this is unlikely as the improvements in goodness of fit and class stability by including more classes were small after inclusion of a sixth class (online supplemental table 3). Second, there is a healthy volunteer bias in the UKB, meaning these individuals may not be truly representative of the UK-based T2D population. A higher proportion of T2D patients may therefore be assigned to non-reference classes in a more representative UK sample. Cohorts that do not rely on individual-level recruitment, such as the Clinical Practice Research Datalink, should be used to validate our results. Third, T2D diagnosis criteria have changed substantially over the past two decades, with increased population-wide T2D screening and monitoring. This could introduce cohort-specific effects related to the start date of medical records in the UKB. Fourth, despite the large number of participants in our study, only 8% and 4% had prevalent and incident MDD respectively, limiting our ability to fully determine the relationship between HbA1c class trajectories and MDD. Finally, due to limited sample size for individuals of non-European ancestry, and the lack of availability for well-powered genetic studies in individuals in this group, we excluded individuals who were non-European. As there is known heterogeneity by ancestry for T2D disease progression and outcomes,^40^ further studies should focus on extending these methods to individuals of non-European ancestry.

In conclusion, six classes with distinct HbA1c trajectories were identified in 12,435 individuals of European ancestry with T2D, using the UKB linked to primary care data. Participants in non-reference classes (23%) had increased risk of developing T2D complications, including all-cause mortality. Younger age at T2D diagnosis and lower healthcare utilization prior to diagnosis were identified as important risk factors for following an atypical trajectory. This suggests that the risk of developing complications is related to lower healthcare utilization prior to T2D diagnosis. Additionally, although all individuals in this study that followed an atypical trajectory were at higher risk of developing complications, we identified ∼7% of participants (Classes C, D, E) who displayed highly accelerated T2D progression, as defined by a greater degree of medication transition and a higher risk of developing T2D complications and all-cause mortality. In summary, this study shows that participants who diverge from the typical T2D HbA1c trajectory can be identified as targets for intensive intervention.

## Supplementary material

10.1136/bmjdrc-2024-004826 online supplemental file 1

10.1136/bmjdrc-2024-004826 online supplemental file 2

10.1136/bmjdrc-2024-004826 online supplemental file 3

10.1136/bmjdrc-2024-004826 online supplemental file 4

10.1136/bmjdrc-2024-004826 online supplemental file 5

10.1136/bmjdrc-2024-004826 online supplemental file 6

10.1136/bmjdrc-2024-004826 online supplemental file 7

10.1136/bmjdrc-2024-004826 online supplemental file 8

10.1136/bmjdrc-2024-004826 online supplemental file 9

10.1136/bmjdrc-2024-004826 online supplemental file 10

10.1136/bmjdrc-2024-004826 online supplemental file 11

## Data Availability

The data that support the findings of this study are available from UKB, but restrictions apply to the availability of these data, which were used under license for the current study and therefore are not publicly available.
