# Supplementary material for: Latent class growth mixture modeling of HbA1C trajectories identifies individuals at high risk of developing complications of type 2 diabetes mellitus in the UK Biobank
Source: BMJ Open Diabetes Res Care. 2025 Sep 8;13(5):e004826. doi: 10.1136/bmjdrc-2024-004826 (PMC12421182; doi:10.1136/bmjdrc-2024-004826)
Supplement: online supplemental file 10 [file bmjdrc-13-5-s010.docx]

**Supplementary Figure 1.** Methods flow chart. BIC, Bayesian information criteria; APPA, average posterior probability of assignment; LMR-LRT, Lo-Mendell-Rubin likelihood ratio test.

**
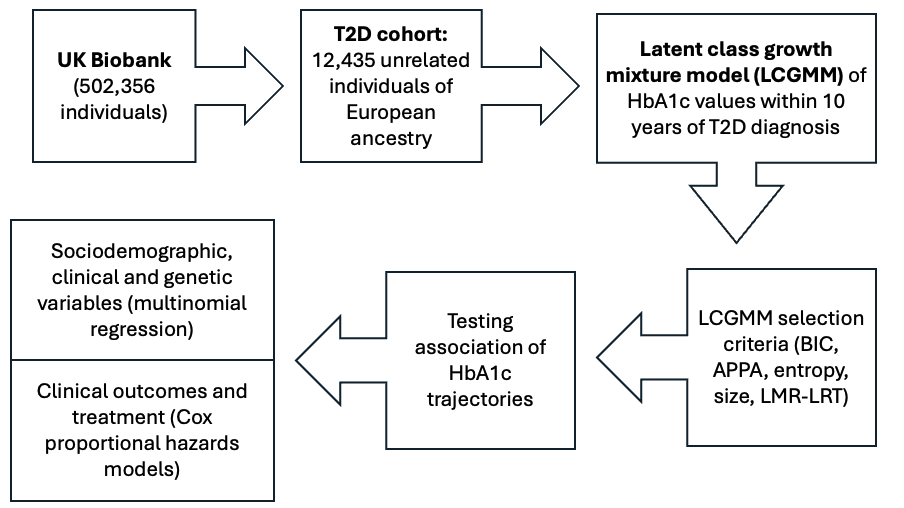
**

Supplementary Figure 2. Upset plot of T2D diagnostic criteria met.


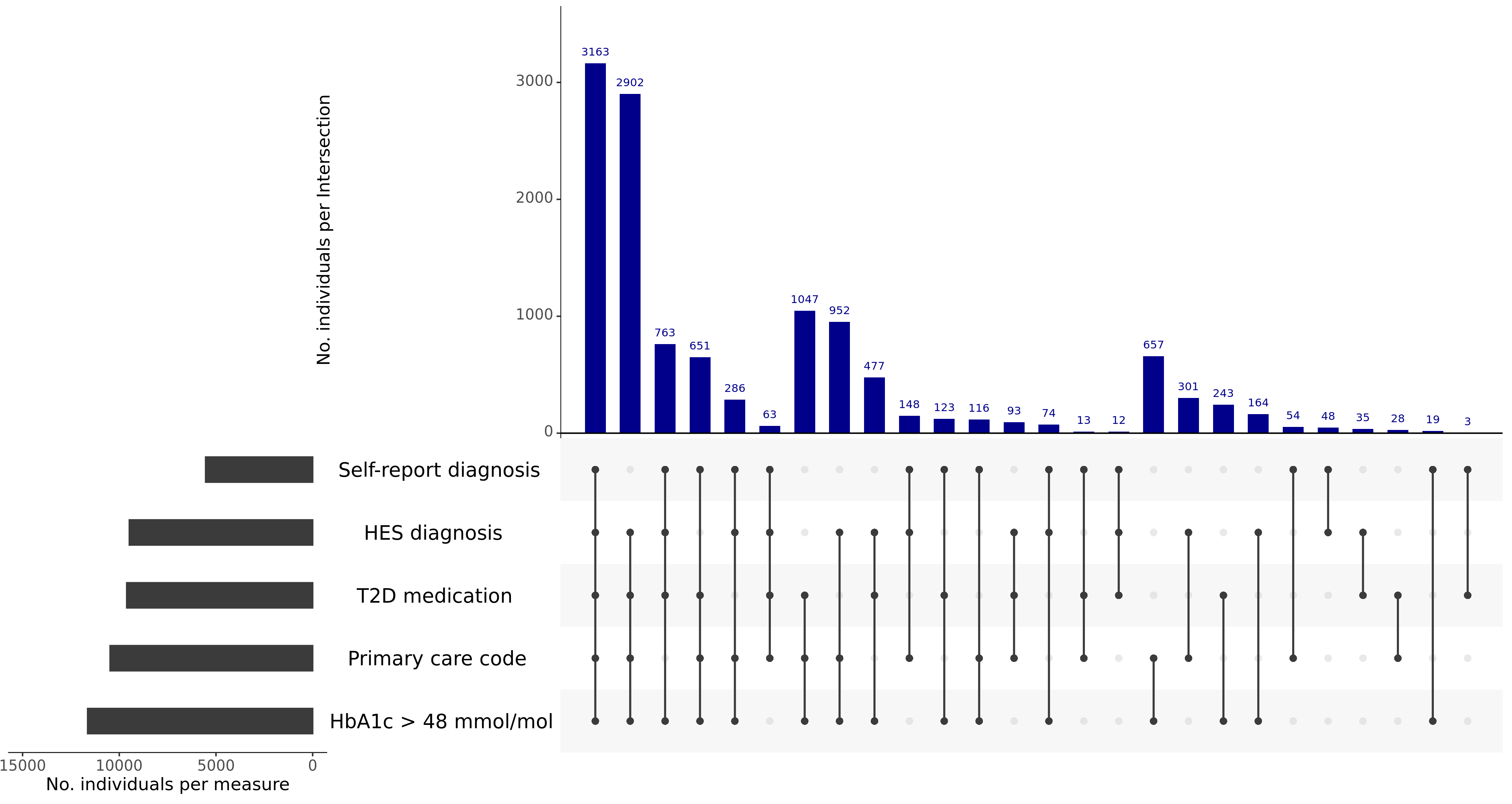


Supplementary Figure 3: Descriptives of the six-classes in the latent class growth mixture model, ordered by the predicted HbA1c level at diagnosis.

| Class | Figure | No. T2D cases (%) | Descriptive name | Information |
| --- | --- | --- | --- | --- |
| A | 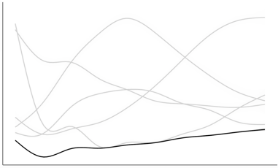 | 9550 (76.8%) | Low and stable (Reference) | Lowest HbA1c levels at diagnosis, which increased only slightly over the 10-years of follow-up. |
| B | 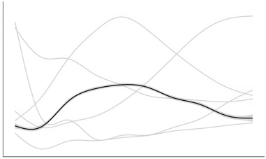 | 1130 (9.06%) | Low parabolic | Low HbA1c levels at diagnosis, increasing to a 70 mg/dL peak at five years, followed by a return to levels near those at diagnosis at 10 years. |
| C | 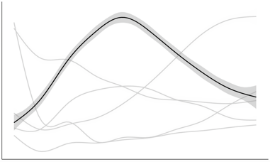 | 245 (1.97%) | High parabolic | Low HbA1c levels at diagnosis, increasing to a high peak (>90 mg/dL) at five years, followed by a return to lower levels at 10 years. |

| Class | Figure | No. T2D cases (%) | Descriptive name | Information |
| --- | --- | --- | --- | --- |
| D | 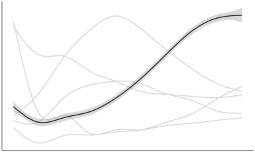 | 315 (2.53%) | Steep increase | Low HbA1c levels at diagnosis, which increased rapidly after year 2. |
| E | 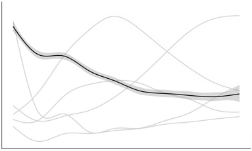 | 326 (2.62%) | Slow decrease | High HbA1c levels at diagnosis, which decreased slightly over 10 years but remained well above the baseline group. |
| F | 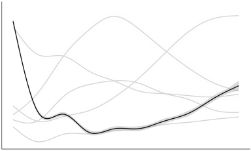 | 874 (7.03%) | Rapid decrease | High HbA1c levels at diagnoses, which decreased rapidly to levels like the baseline group, but with a further increased after 6 years. |
