# Supplementary material for: Latent class growth mixture modeling of HbA1C trajectories identifies individuals at high risk of developing complications of type 2 diabetes mellitus in the UK Biobank
Source: BMJ Open Diabetes Res Care. 2025 Sep 8;13(5):e004826. doi: 10.1136/bmjdrc-2024-004826 (PMC12421182; doi:10.1136/bmjdrc-2024-004826)
Supplement: online supplemental file 11 [file bmjdrc-13-5-s011.docx]

**Supplementary Information**

**Methods S1. Sample quality control**

Standard sample quality control for the entire UKB cohort was applied using previously described methods[1]. A kinship threshold of with a Kinship threshold of 0.044 was used to identify related sets of individuals using the GreedRelated R package as part of the *UKBKings* R package [2,3], and only one individual was retained from each related set. Genetic ancestry was determined using the *UKBKings* R package, which uses a previously validated method for ancestral determination [2]. Genetic sex was determined as outlined previously and provided by the UK Biobank [1]. Briefly, a combination of X chromosome heterozygosity and Y chromosome genotyping signal intensity were used to determine genetic sex. Individuals who had a genetic sex that was misaligned with their self-reported sex, indeterminable sex, or showed evidence of sex chromosome aneuploidy were removed from further analyses.

**Methods S2. Quality control for HbA1c measurements**

All HbA1c values were converted from Diabetes Control and Complications Trial units (percentage units; DCCT) to International Federation of Clinical Chemistry and Laboratory Medicine values (mmol/mol units; IFCC). HbA1c values between 15 and 20 before transformation were removed, as it is unclear whether they represent high DCCT values or low IFCC values. As HbA1c values from the UKB assessments are systematically lower than those in the primary care records, the UKB measures were calibrated using the following equation: [calibrated HbA1c] = 0.9696[raw HbA1c] + 3.3595.

**Methods S3. LCGMM specification and computation time**

The Latent Class Growth Mixture Model was generated using the default variance-covariance structure applied by the *lcmm* R package [4]. The model was allowed a maximum of 200 iterations for convergence, and 100 random start points for the model were used to avoid solutions which arrive at local maxima [5]. Models were computed in parallel using 50 CPU cores, and a maximum of 48 hours was allowed for convergence. Due to computational limitations, only a linear time term was included as a random effect. The six-class solution took 42.7 hours to converge, and the seven-class solution did not converge within the allotted time frame.

To select the optimal number of classes, we started by modelling the sample as one homogeneous population and then added one class at a time. We required that a model with *k* classes must meet the following criteria to be considered for selection as the final model compared to the less complex, *k* – 1 class solution:

1) each class in the model contains a minimum of 200 individuals,

2) the Bayesian information criteria (BIC) is lower by at least 100,

3) the average posterior probability of assignment (APPA) exceeds 0.70 for all classes [6],

4) the model has improved goodness of fit compared to the less complex model according to the Lo-Mendell-Rubin Adjusted Likelihood Ratio test (p < 0.05) [7],

5) the model has a relative entropy >0.8, and

6) the model has a mismatch value close to zero for all classes [6].

For Figure 1, The characteristics of the HbA1c trajectories within each cluster were described using visualizations generated with general additive model (GAM) smoother applied to the data for each cluster. These visualizations provide an intuitive representation of within-cluster trends but may differ slightly from the trajectories estimated by the LCGMM model due to the increased flexibility of the GAM.

**Methods S4. Polygenic score generation**

PRSCS, via GenoPredPipe was used to generate polygenic scores for three traits: Body mass index, Type 2 diabetes (T2D), and major depressive disorder (MDD) using GWAS summary statistics containing individuals of European ancestry that had minimal sample overlap with the UK Biobank [8–12].

**Methods S5. Polygenic score validation**

Polygenic score validation was performed in all available unrelated individuals in the UK Biobank of European ancestry for BMI, and in individuals who met these criteria and had primary care record data available for T2D and MDD.

BMI was defined using UK Biobank field 21001. T2D status was derived using the cohort outlined by the primary text, and Individuals who did not meet any criteria for T2D diagnosis were controls. MDD was derived solely in individuals with primary care record data available, according to previously described methods [13].

Linear regression was used for BMI, and logistic regression was used for T2D status and MDD status, to determine the association of the polygenic scores with their respective traits, corrected for age at measurement or index date, sex, and the first six principal components of ancestry.

**Methods S6. Proportional hazards assumption sensitivity analyses**

All exposure–outcome pairs were first assessed for non-proportional hazards using *cox.zph* function from the *survival* R package, which tests whether the scaled Schoenfeld residuals are uncorrelated with time [14]. The resulting p-values from these tests were adjusted for multiple testing using the Holm–Bonferroni method, and any exposure–outcome pair with an adjusted p < 0.05 were deemed to violate the proportional hazards assumption.

For the class membership - outcome models which contained at least one exposure that violated the proportional hazards assumption, we fitted Royston–Parmar models using the proportional odds scale and incorporating a five-knot spline to capture time-dependent effects, using the *flexsurv* R package [15]. These models had the same covariate structure as described for the Cox models as outlined in the main manuscript methods text.

**Methods S7. Definitions of medication progression phenotypes**

Two medication phenotypes were included as measures of T2D disease progression rate [16]. Firstly, “Progression to combination therapy” was defined as the time between T2D diagnosis and the first prescription record for a non-metformin medication and calculated for individuals who had at least one prescription record for metformin. Secondly, “Progression to insulin” was calculated for all individuals as the time from T2D diagnosis to the first prescription of insulin or an insulin-containing medication. Full code lists for T2D medications were published previously[17].

**Results S1. Polygenic score validation**

All three polygenic scores were robustly associated with their respective outcome traits (Supplementary information, Table 1).

Supplementary Information, Table 1. The association of polygenic scores with their relevant traits. R^2^ is reported for linear regression, and Nagelkerke’s pseudo-R^2^ is reported for logistic regression.

| **Polygenic score** | **R^2^** | **p-value** |
| --- | --- | --- |
| Body mass index | 0.081 | < 1x10^-200^ |
| Type 2 diabetes mellitus | 0.040 | < 1x10^-200^ |
| Major depressive disorder | 0.0073 | 5.59x10^-156^ |

**Results S2. Proportional hazards assumption violation and Royston-Palmer models**

Of the 15 Cox models used to analyse class membership – outcome associations, eight included at least one exposure that violated the proportional hazards assumption according to the Schoenfeld residual tests and were investigated further (Supplementary Table 6). According to the Royston-Palmer models, although the association between class B membership and the risk of diabetic retinopathy lost statistical significance (OR: 1.14 [1.01–1.29], p = 0.11), all other associations remained statistically significant (Supplementary Tables 4 and 7). Therefore, the violations of the proportional hazards assumption did not substantially change the conclusions drawn from the Cox proportional hazards models presented in the main manuscript.

**References**

1 Bycroft C, Freeman C, Petkova D, *et al.* The UK Biobank resource with deep phenotyping and genomic data. *Nature*. 2018;562:203–9. doi: 10.1038/s41586-018-0579-z

2 Hanscombe K. kenhanscombe/ukbkings. 2024.

3 Sam Choi / GreedyRelated · GitLab. GitLab. 2020. https://gitlab.com/choishingwan/GreedyRelated (accessed 27 May 2025)

4 Proust-Lima C, Philipps V, Liquet B. Estimation of Extended Mixed Models Using Latent Classes and Latent Processes: The R Package lcmm. *Journal of Statistical Software*. 2017;78:1–56. doi: 10.18637/jss.v078.i02

5 Hipp JR, Bauer DJ. “Local Solutions in the Estimation of Growth Mixture Models”: Correction to Hipp and Bauer (2006). *Psychological Methods*. 2006;11:305–305. doi: 10.1037/1082-989X.11.3.305

6 Lennon H, Kelly S, Sperrin M, *et al.* Framework to construct and interpret latent class trajectory modelling. *BMJ Open*. 2018;8:e020683. doi: 10.1136/bmjopen-2017-020683

7 Lo Y, Mendell NR, Rubin DB. Testing the number of components in a normal mixture. *Biometrika*. 2001;88:767–78. doi: 10.1093/biomet/88.3.767

8 Locke AE, Kahali B, Berndt SI, *et al.* Genetic studies of body mass index yield new insights for obesity biology. *Nature*. 2015;518:197–206. doi: 10.1038/nature14177

9 Scott RA, Scott LJ, Mägi R, *et al.* An Expanded Genome-Wide Association Study of Type 2 Diabetes in Europeans. *Diabetes*. 2017;66:2888–902. doi: 10.2337/db16-1253

10 Wray NR, Ripke S, Mattheisen M, *et al.* Genome-wide association analyses identify 44 risk variants and refine the genetic architecture of major depression. *Nat Genet*. 2018;50:668–81. doi: 10.1038/s41588-018-0090-3

11 Ge T, Chen C-Y, Ni Y, *et al.* Polygenic prediction via Bayesian regression and continuous shrinkage priors. *Nat Commun*. 2019;10:1776. doi: 10.1038/s41467-019-09718-5

12 Pain O, Al-Chalabi A, Lewis CM. The GenoPred Pipeline: A Comprehensive and Scalable Pipeline for Polygenic Scoring. 2024;2024.06.12.24308843.

13 Fabbri C, Hagenaars SP, John C, *et al.* Genetic and clinical characteristics of treatment-resistant depression using primary care records in two UK cohorts. *Molecular Psychiatry*. 2021;26:3363. doi: 10.1038/s41380-021-01062-9

14 Therneau TM, Grambsch PM. *Modeling Survival Data: Extending the Cox Model*. New York, NY: Springer 2000.

15 Jackson C. flexsurv: A Platform for Parametric Survival Modeling in R. *Journal of Statistical Software*. 2016;70:1–33. doi: 10.18637/jss.v070.i08

16 Fonseca VA. Defining and characterizing the progression of type 2 diabetes. *Diabetes Care*. 2009;32 Suppl 2:S151-156. doi: 10.2337/dc09-S301

17 Gillett AC, Hagenaars SP, Handley D, *et al.* The impact of major depressive disorder on glycaemic control in type 2 diabetes: a longitudinal cohort study using UK Biobank primary care records. *BMC Medicine*. 2024;22:211. doi: 10.1186/s12916-024-03425-9
